# Cryopreservation of oil palm (*Elaeis guineensis* Jacq.) polyembryoids via encapsulation–desiccation

**DOI:** 10.1007/s13205-019-1997-9

**Published:** 2019-12-03

**Authors:** Sharrmila Rengeswari Palanyandy, Saikat Gantait, Sreeramanan Subramaniam, Uma Rani Sinniah

**Affiliations:** 1 Department of Crop Science, Faculty of Agriculture, Universiti Putra Malaysia, Serdang, 43400 Selangor Malaysia; 2 Crop Research Unit (Genetics and Plant Breeding), Bidhan Chandra Krishi Viswavidyalaya, Mohanpur, Nadia, West Bengal 741252 India; 3 School of Biological Sciences, Universiti Sains Malaysia, Minden Heights, 11800 Penang Malaysia

**Keywords:** Developmental stage, Dehydration, Preculture, Regrowth, Scanning electron microscopy

## Abstract

The current report assesses the efficiency of encapsulation–desiccation protocol to cryopreserve oil palm (*Elaeis guineensis* Jacq.) polyembryoids. Specifically identified polyembryoids, comprising of haustorium and torpedo-shaped structures, were encapsulated [comprising 3% (w/v) sodium alginate and 100 mM CaCl_2_]. Calcium alginate-encapsulated and sucrose-precultured polyembryoids were subjected to different spans of desiccation in a laminar air-flow cabinet, followed by freezing in liquid nitrogen. The effect of sucrose preculture (with gradual exposure to 0.3, 0.5, 0.75 and 1 M for 7 days) and dehydration periods (0–10 h) under sterile air-flow on post-freezing survival and regrowth of encapsulated polyembryoids were studied. Cryopreserved and thawed polyembryoids (initially precultured in sucrose, followed by 9 h air-desiccated to 23.3% moisture content) displayed the highest survival percentage (73.3%) and regeneration (of shoot, root and secondary somatic embryo) on Murashige and Skoog regrowth medium containing sucrose (0.3–1 M) and 0.2 mg/l 2,4-dichlorophenoxy acetic acid. In addition, ultrastructural study using scanning electron microscopy exhibited successful revival of cryopreserved polyembryoids, owing to retention of cellular membrane stability through optimized and protected (encapsulated) desiccation. The present study thus substantiates the potential of this encapsulation–desiccation procedure in cryopreservation of oil palm polyembryoids for long-term conservation programs.

## Introduction

Oil palm (*Elaies guineensis* Jacq.) is a tropical plant, which originates from the Gulf of Guinea in West Africa, hence popularly known as the African oil palm. It is a perennial crop with a productive life of longer than 50 years; however, economically, the life span is about 20–25 years. Palm oil is now a primary source of viable and renewable raw material for the world’s food, oleo chemical and biofuel industries. It is currently the world’s second most highly consumed oil. Palm oil production in Malaysia, the second biggest grower after Indonesia, for the year 2019 is anticipated to cross 24 million tonnes as compared to ~ 20 million tonnes that was produced in 2018 (http://bepi.mpob.gov.my/index.php/en/).

Vegetative propagation of oil palm by tissue culture has significant advantages over conventional breeding (Sogeke 1998). Tissue culture is extensively utilized for mass propagation of oil palm mainly by large companies for their own use and at a pilot scale. Theoretically with the aid of tissue culture, the uniformity of the planting materials can be maintained, thus leading to an increase in yield of up to 20% in contrast to seedlings. However, the tissue culture technique for oil palm is quite complex and time consuming. In oil palm, the main complication that impedes the development of an economically sustainable propagation system is the non-uniform growth of tissue-cultured materials in culture jar, as well as low frequency of embryogenesis and plantlet production (Dumet et al. 1993a; Gantait et al. 2017). In addition, the time frame needed for the formation of complete plantlets from cell suspension culture also takes very long duration which is around 2–3 years. Often within a single culture vessel, various stages of development can be observed (Gantait et al. 2015). Hence, the ability to identify the best stage for conversion into plantlet is necessary to produce uniform seedlings. In oil palm, detailed description of the stages from cell suspension culture to plantlet formation, and identification of optimal stage for conservation were well documented (Palanyandy et al. 2013). However, upon obtaining the best stage of development, it is necessary to establish methods to conserve the propagules for future use. To store the samples for later use, short- or long-term, the simplest approach would be to employ cryopreservation techniques.

Nevertheless, in the past two decades, cryopreservation technique has gained much popularity as a storage technique for difficult-to-store materials. It is also often a technique that is used for long-term conservation of genetic recourses of intermediate and recalcitrant materials. Cryopreservation is an attractive alternative for the storage of plant material at ultra-low temperature (− 196 °C); specifically, the temperature of liquid nitrogen (LN). At this temperature, cell division and all other biological activities get completely arrested. According to Towill (1991), allocation of cells from room temperature to − 196 °C should be done in such a manner that the viability of the stored material is maintained, so that their biological activities and growth can be resumed following thawing and transfer to regrowth medium. Whilst some cryopreservation methods are dependent upon the use of expensive cryogenic facilities, yet there are some techniques that involve simplified procedures, which permit direct immersion of plant material in LN for cryopreservation. For instance, the encapsulation–desiccation procedure (Dereuddre et al. 1990) is one of such techniques in which the plant material is coated with alginic acid solution and desiccated prior to LN exposure (Gantait et al. 2017).

Generally, naked tissues are very sensitive towards mechanical injuries due to changes in the culture conditions. Mechanical injuries occur easily during handling and transfer process, especially due to usage of forceps. Continual exposure in same media for certain duration also can lead to desiccation of tissues. Alginate encapsulation and sucrose preculture was successfully utilized in many studies to minimize the losses owing to handling as well as desiccation treatment (Gantait et al. 2017). Although there exists some limited reports on alginate encapsulation (Inpuay and Te-chato 2012; Mariani et al. 2014; Palanyandy et al. 2015), however, comprehensive published work on oil palm for cryopreservation is scarce till date. It is hypothesized that encapsulation of oil palm polyembryoids, followed by a degree of desiccation will make the embryoids latent so that growth would reinstate only after the moisture content is elevated. This method (encapsulation) will protect the tissues from any mechanical damage as it also will ease handling while in storage, either for short-term or long-term. Earlier studies have shown that artificial seeds treated with sucrose preculture gave better response since sucrose protects the tissues and nucleic acid from inactivation and also acts as an osmoticum agent that dehydrates the tissues and stabilizes the membrane (Towill 1991). In addition, encapsulation helps to protect tissue from direct exposure to sucrose preculture, since increasing levels of sucrose concentration may lead to toxicity. Explants are encapsulated in alginate beads, pre-grown in liquid medium enriched with sucrose, partially desiccated to a moisture content, then frozen rapidly (Engelmann 1997). The reduction of the moisture content (MC) of encapsulated explants to a minimal level is a necessary step for successful cryopreservation (Gonzalez-Arnao and Engelmann 2006). This aim could be achieved by combining the preculture of the encapsulated explants in sucrose-containing medium with their subsequent dehydration in silica gel or under sterile air-flow.

Therefore, the objective of this study was to assess the effects of sucrose preculture and desiccation on encapsulated and naked (non-encapsulated) oil palm polyembryoids, and also to assess the effects of LN exposure on encapsulated oil palm polyembryoids to obtain optimum survival percentage for storage.

## Materials and methods

### Selection of polyembryoids

Cell aggregates smaller than 500 µm in size were removed by sieving and placed back in liquid medium for aggregation. Meanwhile, cell aggregates more than 500 µm in size were transferred to plant growth regulator (PGR)-free Murashige and Skoog (MS) (Murashige and Skoog 1962) semi-solid medium, which consist of 30 g/l sucrose, vitamins and 3.5 g/l Gelrite^®^. The cultures were maintained at 25 °C under standard cool fluorescent tubes (Philips Lifemax, PHILIPS, Indonesia) at 60 µmol/m^2^/s irradiance and 16 h photoperiod. Continuous subculture was done in MS semisolid medium and the development of aggregates, until the formation of embryoids, was observed periodically to identify and collect polyembryoids that have reached the optimal developmental stage for cryopreservation (following Gantait et al. 2015).

### Encapsulation of polyembryoids

The selected oil palm polyembryoids were isolated aseptically at their appropriate synchronous stage. To develop alginate beads, each of the separated polyembryoids was dipped in calcium-free liquid MS basal medium with 3% (w/v) sodium alginate (Gonzalez-Arnao and Engelmann 2006). The aliquotes containing individual polyembryoids were then dropped gently with a pipette into 100 mM CaCl_2_ solution that in turn induced the polymerization of alginate, thus resulting in the formation of coats around the polyembryoids. After the last bead (encapsulated polyembryoid) had formed, these were then kept in CaCl_2_ solution for 20–30 min to ensure optimum polymerization. These spherical beads typically measured 4–5 mm in diameter. Recovery of the beads was effectuated by draining the solution, followed by rinsing the beads twice with sterile MS basal medium, and subsequent blotting using filter paper in petri dishes.

### Sucrose preculture

The alginate beads, each containing individual polyembryoid, were transferred (for every alternative day) to the sucrose preculture medium containing increasing levels of sucrose (0.3, 0.5, 0.75 and 1 M) for 7 consecutive days, using gradual sucrose dehydration technique. Similarly, naked (non-encapsulated) polyembryoids were exposed to sucrose preculture treatment. Beads containing the explants in sucrose medium were placed in gyratory shaker at 80 rpm. Initially, the pH of the medium was adjusted to 5.7 with 0.1 M NaOH or HCl followed by sterilization at 121 °C for 20 min. At the same time, naked as well as encapsulated polyembryoids, that were not exposed to sucrose preculture, were considered as control (reference) treatments.

### Desiccation

Following osmoprotection using sucrose preculture, beads were rapidly surface dried on sterile filter paper to remove any remaining liquid medium and subjected to physical dehydration under the air current of a laminar air-flow (LAF) cabinet for different desiccation hours (DHs), which were 0–10 h at 1 h intervals. After each desiccation period, the beads were subjected for MC determination, and subsequently cultured aseptically on PGR-free MS semi-solid medium. Regular monitoring was carried out on the early developmental changes. The percentage of polyembryoids that swelled and enlarged in size was recorded as ‘viable’ after 2 weeks of culture. Meanwhile, the percentage of polyembryoids that turned green was recorded as ‘survived’ after 4 weeks of culture. Furthermore, morphological characteristics such as number of leaf, root, secondary somatic embryo (SSE), and callus were recorded after 50 days of culture.

### Determination of moisture content

The MC of polyembryoids was determined with the aid of the ‘low constant temperature oven method’ (ISTA 2005). The polyembryoids were placed on a pre-weighed aluminium boat; the weight of boat with polyembryoids was noted and then the boat and polyembryoids were placed in the oven at 103 °C for 16 h. Upon removal from the oven, the polyembryoids were placed in desiccators to cool down. After 30 min, the boat with the dried polyembryoids was reweighed. The MC was measured in terms of loss of weight and presented as percentage of initial fresh weight. The MC% of polyembryoids was determined by the following formula:

$${\text{MC}}\% = \left[ {\left( {W_{2} - W_{3} } \right)/\left( {W_{2} - W_{1} } \right)} \right] \times 100,$$

where *W*_1_ = weight of aluminium boat, *W*_2_ = weight of aluminium boat + polyembryoids before drying, *W*_3_ = weight of aluminium boat + polyembryoids after drying.

### Liquid nitrogen exposure and post-freezing regrowth

After dehydration, the beads were recovered and placed in 1 ml polypropylene sterile cryotubes to be immersed in LN. After 1 h, the samples were recovered from LN and rapid warming (thawing) is performed, wherein the cryotubes were stirred in a water bath for 2–3 min at 40 °C. Subsequently, the thawed polyembryoids were isolated and cultured on recovery media. For recovery, the embryos were cultured for 1 week on MS medium containing 0.3 M sucrose and 0.2 mg/l 2,4-dichlorophenoxy acetic acid (2,4-D), and again for 2 weeks on a medium containing 0.1 M sucrose and the same concentration of 2,4-D in dark. They were then transferred onto standard MS medium devoid of PGRs. The polyembryoids were kept in the dark for 3 weeks prior to exposure to light at 25 °C under standard cool fluorescent tubes (Philips Lifemax, PHILIPS, Indonesia) at 180 µmol/m^2^/s irradiance and 16 h photoperiod.

### Scanning electron microscopic (SEM) observation

During post-freezing regrowth, polyembryoids were prefixed with 4% glutaraldehyde in 0.1 M sodium cacodylate buffer (pH 6.8) at 4 °C for 48 h. Samples were then post-fixed in 1% osmium tetroxide at 4 °C for 2 h. The samples were then cleansed thrice with 0.1 M sodium cacodylate buffer (pH 6.8) at 30 min each. Thereafter, they were progressively dehydrated in a graded acetone series, and dried for 30 min at the critical point in Leica EM CPD 030 Critical Point Dryer (Leica, Germany) (using CO_2_ as the transient fluid). Ultimately, the polyembryoids were attached on stubs using colloidal silver and then, coated with gold particles by Polaron E5100 sputter coater (Polaron, USA), and further examined under Leo 1455 VPSEM (Leo, USA) SEM.

### Statistical analysis

The experiments were organized in a completely randomized design. All the experiments were replicated thrice, using 50 samples for each replicate. The data collected were analysed using analysis of variance (ANOVA) and significant differences among the treatments were compared based on Duncan’s multiple range test (Duncan 1955) at *P* ≤ 0.05 with the aid of SPSS (Version 11, SPSS Inc. Chicago, USA) software package. All percent data were transformed to square root values before subjected to ANOVA.

## Results

### Effect of sucrose preculture

During sucrose preculture experiment, 100% viability was observed in the cases of encapsulated and sucrose precultured (E/SP), encapsulated and non-precultured (E/NP) and naked and non-precultured (N/NP) polyembryoids, without desiccation in LAF. However, only 63.3% viability was obtained for naked and sucrose precultured (N/SP) polyembryoids without desiccation (Table 1). Based on the results obtained for survival percentage, 100% success was observed only for encapsulated and sucrose precultured (E/SP) polyembryoids, whereas survival percentage obtained for all the other treatments was less, without desiccation (DH 0). In addition, the results showed with the increase in DH, the viability and survival percentage reduced for all the treatments. The encapsulated polyembryoids which were precultured on sucrose displayed a slower trend in reduction of viability and survival percentage with the increase DH when compared to other treatments. Meanwhile, a drastic reduction in viability and survival percentage was noticed when DH was increased in case of naked and sucrose precultured polyembryoids (Table 1). The morphological observation on growth and development (Table 2) also showed a higher number of leaves, roots, SSE and calli for encapsulated and sucrose precultured polyembryoids, irrespective of desiccation durations. However, the results also exhibited that there were significant difference in growth and development especially for both the naked and encapsulated polyembryoids, before and after sucrose preculture.

**Table 1:** Viability and survival of encapsulated and naked oil palm polyembryoids with sucrose preculture and desiccation treatment after 2 and 4 weeks of culture

	DH 0	DH 1	DH 2	DH 3	DH 4
Viability (%)					
E/SP	100.0a	86.7a	80.0a	66.7a	63.3a
N/SP	63.3b	33.3c	23.3c	13.3c	13.3c
E/NP	100.0a	80.0ab	63.3b	43.3bc	40.0bc
N/NP	100.0a	76.7b	63.3b	46.7b	43.3b
Survival (%)					
E/SP	100.0a	83.3a	76.7a	63.3a	56.7a
N/SP	60.0c	30.0c	20.0c	10.0c	6.7c
E/NP	93.3ab	73.3b	56.7bc	40.0bc	36.7b
N/NP	83.3b	73.3b	60.0b	43.3b	36.7b

Mean values (of individual charater) in the same column having the different letters are significantly different at *P* ≤ 0.05 based on Duncan Multiple Range Test (Duncan 1955)

*E/SP* encapsulated/sucrose precultured, *N/SP* naked/sucrose precultured, *E/NSP* encapsulated/non-precultured, *N/NSP* naked/non-precultured, *DH* desiccation hour

**Table 2:** Effect of sucrose preculture and desiccation on number of leaf, root, secondary somatic embryo (SSE), and callus formed from oil palm polyembryoids after 50 days in culture

	DH 0	DH 1	DH 2	DH 3	DH 4
No. of leaf					
E/SP	6.0a	5.3a	4.0a	3.0a	2.3ab
N/SP	2.3c	2.3c	1.7b	1.0b	1.0c
E/NP	3.3bc	4.0b	3.0ab	2.7ab	2.7a
N/NP	4.3b	4.3ab	3.0ab	2.7ab	2.0b
No. of root					
E/SP	3.7a	3.3a	2.3a	1.7a	1.7a
N/SP	1.3c	2.0c	0.7c	1.0b	1.0b
E/NP	1.7bc	2.3bc	1.3ab	1.7a	1.3ab
N/NP	2.3b	2.7b	1.0b	1.3ab	1.3ab
No. of SSE					
E/SP	4.7ab	5.0a	3.7a	2.0ab	2.0ab
N/SP	3.0b	2.0c	1.3c	1.3b	1.3c
E/NP	4.7ab	4.7ab	2.3ab	2.0ab	2.0ab
N/NP	5.7a	4.0b	2.0b	2.3a	2.3a
No. of callus					
E/SP	3.7a	3.3a	2.3b	3.3b	3.3ab
N/SP	2.7c	1.3c	1.3c	1.7c	1.7c
E/NP	3.2ab	3.0b	3.3a	4.0a	4.0a
N/NP	3.0b	3.0b	3.0ab	3.7ab	3.0b

Mean values (of individual character) in the same column having the different letters are significantly different at *P* ≤ 0.05 based on Duncan Multiple Range Test (Duncan 1955)

*E/SP* encapsulated/sucrose precultured, *N/SP* naked/sucrose precultured, *E/NSP* encapsulated/non-precultured, *N/NSP* naked/non-precultured, *DH* desiccation hour

### Effect of desicccation duration on post-freezing survival of polyembryoids

**Fig. 1:**
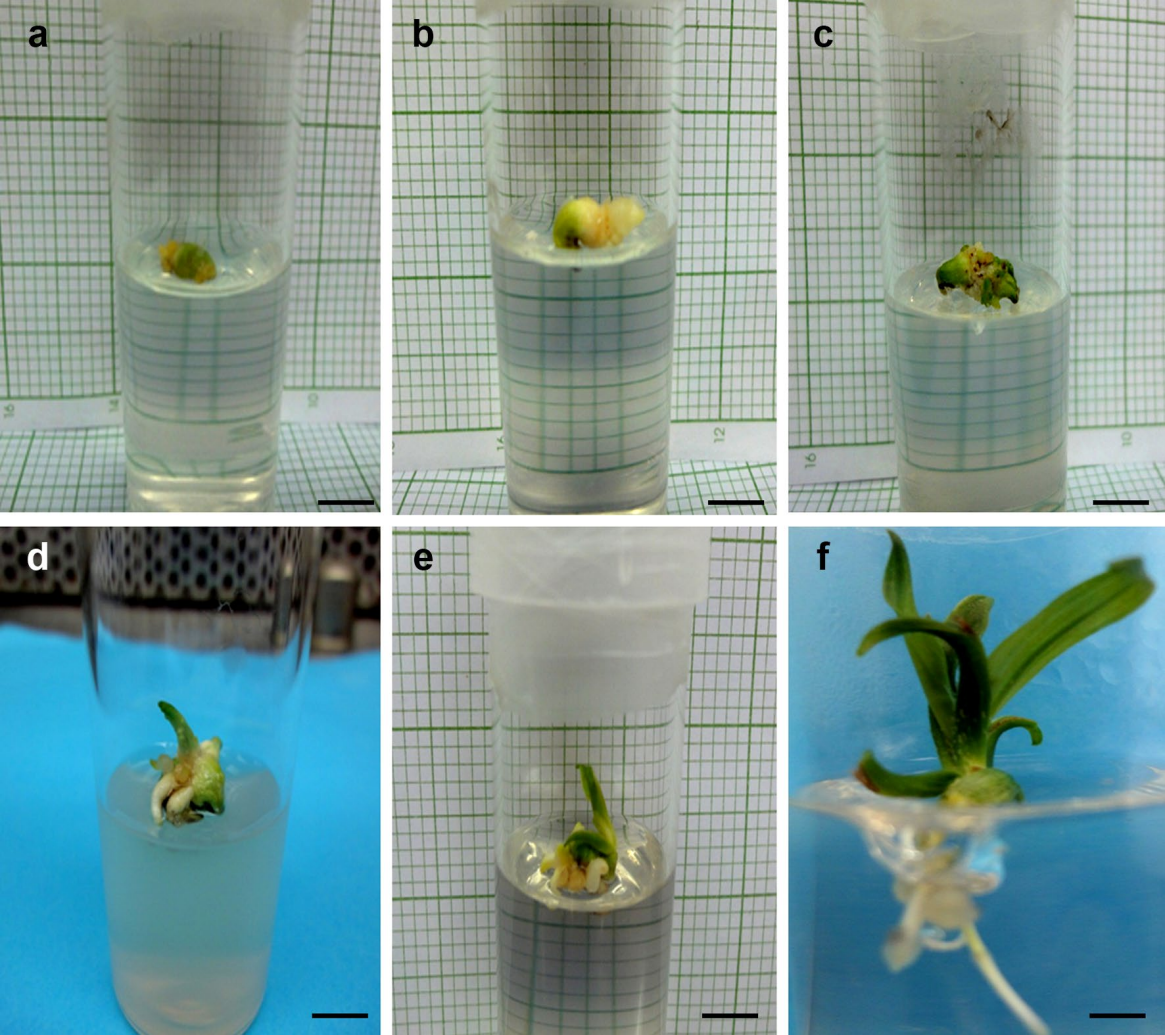
Survival, regrowth and complete plantlet development from 9 h desiccated oil palm polyembryoids following LN exposure, cultured on MS regrowth medium containing sucrose and 0.2 mg/l 2,4-dichlorophenoxy acetic acid. **a** Freshly cultured LN-recovered and thawed polyembryoid wherein encapsulated coat is dried and stuck to polyembryoid (bar = 0.6 cm), **b** polyembryoid started to swell and enlarged in size after 1 week of culture in regrowth medium (bar = 0.6 cm), **c** polyembryoid slowly turned green after 3 weeks of culture (bar = 0.5 cm), **d** polyembryoid ultimately initiated shoot and root formation after 5 weeks of culture (bar = 0.7 cm), **e** plantlet formation after 6 weeks of culture (bar = 0.5 cm), **f** complete plantlets that are amenable for field transfer was obtained around 8 weeks of culture (bar = 0.4 cm)

The initial MC that was obtained for freshly encapsulated polyembryoids was 87% (data not shown). After sucrose preculture, followed by LAF desiccation for 10 h, the MC of the encapsulated polyembryoids declined to 21.3% (Table 3). It is important to determine the optimal MC for successful cryopreservation in order to avoid ice crystallization due to freezing injury, followed by LN exposure and desiccation injury prior to desiccation using LAF. Based on the results obtained, MC around 23.3% (9 h of desiccation) gave the highest survival percentage (73.3%) upon LN exposure (Fig. 1). The results obtained showed that with every increase in DH there was a decrease in MC inversely (Table 3). As stated in methods, after gradual sucrose dehydration for continuous 7 days, the encapsulated polyembryoids were subjected for LAF desiccation for 10 h. After every 1 h, the samples were transferred to cryovials aseptically and exposed to LN. The results showed that the LN-treated samples without desiccation did not survive. All the encapsulated polyembryoids started to turn brown within 5–6 days after being transferred into recovery media, and it fully became black within 30 days, which indicated death. Whereas, without LN treatment the encapsulated polyembryoids resumed their viability after culture in recovery media once desiccated. Significant decrease in viability and survival was recorded with every increase in DH. Contrarily the results that were observed for LN-treated polyembryoids were exactly opposite, whereby with the gradual increase in the DH for 2 h, no viability was observed. The LN-treated polyembryoids started to resume their growth after 3 h desiccation period. Since the purpose of this study was to investigate the basis of the optimal hydration status for cryopreservation of oil palm polyembryoids, further desiccation was conducted until 10 h. A significant decline in survival percentage was observed for the polyembryoids without LN treatment owing to the desiccation duration being increased to 10 h. While for LN-treated polyembryoids the survival percentage was in sync with the increase in DH. Upon exposure to LN, the initial viability and survival percentage that was obtained were 20.0% and 16.7%, respectively, which showed the presence of 43.5% MC after 3 h of desiccation. The survival percentage of cryopreserved polyembryoids continued to increase significantly wherein the highest survival of 73.3% was obtained at 23.3% of MC. Further desiccation showed decrease in survival percentage to 63.3% after 10 h of desiccation. In case of the polyembryoids that were not subjected to LN exposure, the survival percentage decreased to 23.3% when desiccated to 21.3% of MC. It was noticed that the survival percentage of the LN-treated polyembryoids was higher after 8 h of desiccation when compared with control (not treated with LN).

**Table 3:** Effect of desiccation hour and consequent moisture content on survival and regrowth of encapsulated and sucrose-precultured oil palm polyembryoids with and without liquid nitrogen (LN) exposure (after 50 days in culture)

Desiccation hour	Moisture content (%)	Survival (%)		Days to bead break		No. of leaf		No. of root		No. of SSE	
		(− LN)	(+ LN)	(− LN)	(+ LN)	(− LN)	(+ LN)	(− LN)	(+ LN)	(− LN)	(+ LN)
0	67.8a	100.0a	0.0i	11.9d	0.0d	5.6a	0.0e	3.0a	0.0e	7.3ab	0.0f
1	56.7b	80.0b	0.0i	19.3c	0.0d	5.0ab	0.0e	2.7ab	0.0e	7.0abc	0.0f
2	47.4c	73.0c	0.0i	20.0bc	0.0d	4.6ab	0.0e	2.0abc	0.0e	8.3a	0.0f
3	43.5d	66.7d	16.7h	20.5bc	20.1c	3.6bc	1.7d	1.7abc	0.3de	7.3ab	1.3e
4	40.8d	53.3e	23.3g	20.8bc	20.5c	3.0cd	1.3d	2.3abc	1.0cd	6.3bc	2.3de
5	33.7e	53.3e	30.0f	21.0abc	21.1bc	2.7cd	1.3d	1.7abc	1.3bc	5.3cd	3.0d
6	31.6e	46.7f	36.7e	21.3abc	21.6c	2.7cd	1.3d	2.0abc	1.3bc	4.0de	3.7bc
7	28.5f	43.3f	43.3d	22.2abc	22.1ab	2.3cd	2.0cd	1.7abc	1.7abc	4.0de	3.3cd
8	25.9f	36.7g	50.0c	22.4abc	23.7bc	2.3cd	3.0bc	1.3bc	2.0ab	3.7de	4.7ab
9	23.3g	33.3g	73.3a	23.1ab	22.2bc	2.0cd	4.3a	1.0c	2.33a	3.7de	5.3a
10	21.3g	23.3h	63.3b	24.3a	25.1a	1.7d	3.7ab	1.3bc	2.3a	2.3e	3.7bc

Mean values in the same column having the different letters are significantly different at *P* ≤ 0.05 based on Duncan Multiple Range Test (Duncan 1955)

### Effect of desicccation duration on post-freezing days to bead break

**Fig. 2:**
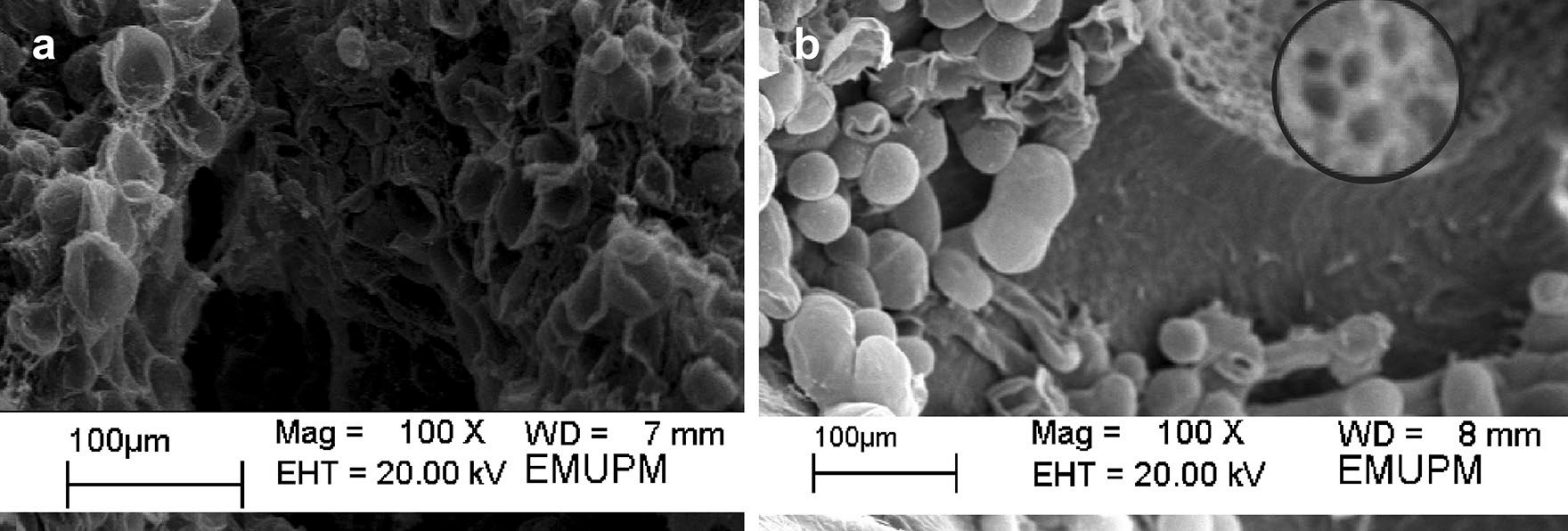
Survival of oil palm polyembryoids following LN exposure, cultured on MS regrowth medium containing sucrose and 0.2 mg/l 2,4-dichlorophenoxy acetic acid. **a** Non-desiccated recovered polyembryoids following LN exposure showing detrimental effects, **b** development of 9 h desiccated encapsulated oil palm polyembryoids following LN exposure showing cellular membrane stability (inset)

Days needed for bead breakage was further examined. Based on the observation, the days needed for bead to break (Table 3) and form plantlets increased as the desiccation duration upsurged either for LN-treated and non-LN-treated polyembryoids. Generally, ~ 12 days were needed for the sucrose precultured polyembryoids to break open the beads and resume their growth without desiccation. Once the polyembryoids were subjected to desiccation, the days needed for beads to break open was significantly delayed. It took ~ 24 days for the beads to break open, once subjected to desiccation for 10 h without LN exposure. There were no significant differences observed in the days needed for LN-treated beads to break open once subjected up to 10 h of desiccation. But there was a significant difference in the days needed to break open for the polyembryoids that were either treated with or without LN. The days needed for the beads to break open was slightly delayed once exposed to LN exposure. A contrasting pattern was observed for the polyembryoids desiccated for 9 h in LAF and exposed to LN, wherein cellular integrity and SSE regrowth were observed to have retained (as evident from SEM study) (Fig. 2b, c). Following this treatment, the beads treated with LN broke open a day earlier when compared to polyembryoids that were not exposed to LN, which was contrary with earlier results. Since, zero survival was observed for LN-treated polyembryoids that were desiccated for 2 h due to presence of high level of MC, no bead was recorded to be broken at this period (Fig. 2a).

### Effect of desicccation duration on post-freezing growth of leaf, root and SSE

Morphological observations were recorded to study the effects of LN on growth of the encapsulated polyembryoids after sucrose preculture and LAF desiccation. The data tabulated in Table 3 showed the number of leaves, roots and SSE that formed after 50 days of culture for both polyembryoids either exposed with or without LN. Based on the survival results, no morphological data could be recorded for the polyembryoids that were encapsulated, precultured (in sucrose), and desiccated for 2 h followed by LN exposure. This was because the beads became dark in colour (black) after 50 days of culture thus indicating death of the polyembryoids. For the control (without LN exposure) polyembryoids, formation of apical buds, leaves, roots and SSE appeared to have reduced with an increase in DH that was evident from SEM observation (Fig. 2a). In case of the control polyembryoids that were exposed to 3 h of desiccation, the results showed that there was a significant reduction in the number of leaves formed. Starting from 4 h of desiccation until 9 h, no significant difference was observed in the formation of leaves. Whereas, for the LN-treated polyembryoids there was no significant difference observed in the formation of leaves starting from 3 h of desiccation to 6 h of desiccation. However, the number of leaves that formed from 7 to 10 h desiccated poltembryoids were observed to be significantly different. For the LN-treated polyembryoids, highest number of leaves formed from those polyembryoids that were desiccated until 9 h in LAF with MC of 23.3% (Fig. 1). Similar trend of results were observed for the number of roots formed after desiccation, whereby there were no significant difference recorded for the formation of roots from polyembryoids desiccated for 2–7 h. It can be clearly observed that roots formation declined with the increase in desiccation duration. Meanwhile, polyembryoids treated with LN formed multiple roots that were significantly different, inspite of applying a different desiccation duration. The highest number of roots (2.3) was observed from those polyembryoids desiccated for 9 h and 10 h. As for the number of SSE that formed without LN exposure, significant difference was recorded till 5 h of desiccation, subsequently followed by non-significant SSE formation from 6 to 9 h of desiccation. The formation of SSE from the LN-treated polyembryoids appeared to be significantly different during the overall desiccation duration (3–10 h). Based on the data, the highest number of SSE was obtained from the LN-treated polyembryoids that were exposed to 9 h of desiccation.

## Discussion

Based on the results obtained from the sucrose preculture experiments, it can be clearly deduced that encapsulated and sucrose precultured polyembryoids exhibited better survival percentage in comparison with that of the non-precultured polyembryoids. This is because sucrose ensures membrane integrity during dehydration and safeguards the stability of isolated proteins in the same way (Carpenter et al. 1993). Generally, once encapsulated and protected with alginate coat, somatic embryos placed on high sucrose concentration medium tends to undergo intensive plasmolysis but without damage (le Roux et al. 2016). Besides that, coating embryos with calcium alginate before progressive plasmolysis, makes it easier to handle them especially after drying under LAF and protects them from injuries caused by excessive dehydration, which eventually results in higher survival percentage after treatment. Thus, based on the results obtained, it can clearly be seen that gradual sucrose preculture gave better survival percentage for oil palm polyembryoids once they were protected with encapsulation coating. During stepwise preculture, the progressive increase in sugar concentration favoured dehydration of encapsulated polyembryoids (Paul et al. 1994). Sucrose pretreatment associated with desiccation was envisaged as a way of inducing a quiescent state in embryos, mimicking the behaviour of true seed. Besides that, true seeds have their own protective seed coat and nutritive accessory tissues (Gray and Purohit 1991), which lacks in somatic embryos. Thus, encapsulation technique appeared to be the best substitute, which would provide nutrients and protective coat for the somatic embryos to grow vigorously, especially in the presence of sucrose, and even after desiccation it would not lose much viability. Superiority of sucrose over other sugars was also reported in cryo-tolerance of oil palm somatic embryo clumps (Dumet et al. 1994). Since encapsulated oil palm polyembryoids, following sucrose preculture, exhibited better survival upon desiccation, it (sucrose preculture) seems to be a promising step for successful storage, either short term or long term. Sucrose, as an effective agent in enhancing dehydration tolerance, has been reported by many researchers (Uchendu et al. 2016; Nakkanong and Nualsri 2018; Yang et al. 2019). As stated earlier, during the sucrose preculture, with the decrease in MC, the amount of dry matter increased considerably. Thus, from this preliminary study, it can be recognized that encapsulation and sucrose preculture is efficient enough for successful storage of oil palm polyembryoids. Meanwhile, exposure of naked polyembryoids to sucrose treatment caused lethal damage, which led to reduction in survival percentage prior to storage; since the naked polyembryoids were directly exposed to increasing levels of sucrose that caused toxic effects to the polyembryoids.

Gradual sucrose preculture, carried out to desiccate the encapsulated polyembryoids, aided in the reduction of MC from 87 to 67.8%. This result indicated that during pretreatment there was an uptake of solutes (sucrose) into the beads, with a concomitant reduction in MC. Further desiccation using LAF would have enhanced the vitrification ability of beads during freezing in LN. The results showed that the viability of LN-treated polyembryoids was directly related to the survival percentage of desiccated polyembryoids. With the existence of high MC, the freezable water present in cells led to ice crystallization during LN exposure which eventually directed to cell death. Therefore, the most critical factor in cryopreservation would be the MC of the encapsulated polyembryoids at the moment of immersion in LN. It was observed that the polyembryoids, following 8 h of desiccation and subsequent LN exposure, survived better than those of the control (not treated with LN). Similar observation was reported by Dumet et al. (1993b) and it can be attributed to differential reactions of clonal nature.

According to Benson (2008), the desiccation phase is vital, since the availability of additional moisture in cell may result in post-freezing chemical, mechanical, and osmotic stresses. It also leads to dysfunction of membrane proteins which would tend to collapse following mechanical stress (Varghese and Naithani 2002). Hence, the span of desiccation period, to attain ideal MC in explants, should therefore be regulated carefully. Limited number of research studies was conducted so far to establish the relationship between gradual decline in ice and glass formation (during freezing) with the enhanced survival of oil palm somatic embryos, following LN exposure (Dumet et al. 1993c). For instance, a high percentage of post-thaw survival was fruitfully documented for oil palm somatic embryos when those were desiccated to 0.5 g H_2_O/g dry weight before freezing (Dumet et al. 1993c). In the present study, the optimum MC that was identified for successful cryopreservation of polyembryoids was 23.3%. LAF desiccation for 9 h proved to be optimum to attain this MC that resulted in highest percentage of survival, supported by morphological evidence. The cryopreservation of oil palm polyembryoids was successfully done using vitrification and droplet-vitrification methods by Suranthran et al. (2012) and Gantait et al. (2015), in which 45% and 68% survival was obtained, respectively. Dumet et al. (1993b) also reported cryopreservation of oil palm by desiccating the embryoids up to a MC of 19–35% with variable success rate. The cryopreservation method that was employed in the present study, as well as the initial plant material used, directly attributed to the differential success rate. High percentage of success rate (73.3% survival) was attained at 23.3% MC, using encapsulation–desiccation method that further established that the encapsulated polyembryoids ensured good percentage of recovery after cryopreservation and led to simultaneous production of plantlets as and when required.

Besides that, encapsulation–desiccation proved to be a better choice over vitrification, since the latter showed a tendency to exert the toxic effects that eventually led to the death of cells. The introduction of plant material to highly concentrated vitrification solution is ideally detrimental owing to the phytotoxic effects of different components and also their collective osmotic effects on cell viability (Towill and Jarret 1992). Moreover, exposure to the vitrification solution for longer duration would result in complete disruption of the plasma membrane (Steponkus et al. 1992). Therefore, these characters are the major reasons that attributed to the reduction in the survival percentage during cryopreservation using conventional vitrification and droplet-vitrification methods in oil palm (Suranthran et al. 2012; Gantait et al. 2015). Hence, as an alternative to vitrification approach, a nontoxic process involving alginate encapsulation and dehydration method was identified as the suitable cryopreservation method for oil palm polyembryoids in this study; wherein, it was distinctly evident that the protective coat provided by encapsulation, protected the polyembryoids from desiccation and freezing injury. Encapsulation–desiccation approach was also used for successful cryopreservation of hop shoot tips (Matinez-Montero et al. 1998), *Ribes ciliatum* (Dumet et al. 2000) and several tropical plant species such as citrus, cassava and potato (reviewed by Gonzalez-Arnao et al. 2008).

It was reported that the duration of air desiccation appeared to be the crucial variable that exerted the maximum influence in several cryopreservation methods used for a range of plant materials including oil palm. In oil palm, post-cryopreservation survival of somatic embryos (precultured in 0.75 M sucrose medium) was significantly enhanced following 12 h (70% survival) and 16 h (80%) desiccation, but without any encapsulation with sodium alginate (Dumet et al. 1993a). On the other hand, following encapsulation, 18 h preculture in 0.75 M sucrose, and 3 h desiccation, survival percentage of in vitro-grown apical meristems of *Ribes* plants was enhanced significantly (Barbara et al. 2005). In the present experiment, lower desiccation time (9 h) was required in LAF to significantly reduce the MC of encapsulated oil palm polyembryoids to ~ 23.3% to obtain the highest survival percentage (73.3%). However, according to Dumet et al. (1993b), it took as high as 16 h desiccation duration to obtain 70% survival after LN exposure, for oil palm somatic embryo. But for the encapsulated and sucrose precultured oil palm polyembryoids, it took lesser desiccation duration (9 h) to obtain optimum MC (23.3%) for a higher survival percentage (73.3. %). Sakai et al. (2000) reported that in an abridged encapsulation–dehydration protocol (using a mixture of 2 M glycerol and 0.4 M sucrose during the encapsulation process and desiccated using silica gel), higher rates of recovery in three plant species (chrysanthemum, mint, and wasabi) was achieved in comparison to that of the typical vitrification methods. It is suggested that this simplified protocol should be applied to oil palm polyembryoids to obtain better success rate. Careful assessment of each step prior to LN exposure is essential to confirm highest viability of cryopreserved plant germplasms. Thus, the potential of different cryopreservation methods should be validated or compared for optimisation of the prime protocol for the conservation of germplasm of interest.

## Conclusion

The experiments performed with oil palm polyembryoids indicated the importance of several parameters, which should be taken into account when developing cryopreservation protocols for somatic embryos such as, stage of explant (polyembryoid), MC, and recovery conditions. The highest survival percentage (73.3%) was attained at 23.3% MC, using encapsulation–desiccation method in this study. It indicated that encapsulated polyembryoids ensured good percentage of recovery after cryopreservation which also led to simultaneous production of plantlets as and when required. Besides, it underlined the vital role of encapsulation for the acquisition of tolerance to sucrose preculture, air desiccation and cryopreservation. These treatments increased their tolerance to desiccation, subsequently reducing the extent of damage, thus indicating the encapsulation–desiccation method as one of the promising cryopreservation techniques for oil palm polyembryoids when compared with other available techniques.

## Abbreviations

DH: Desiccation hour
LAF: Laminar air-flow
LN: Liquid nitrogen
MS: Murashige and Skoog
SEM: Scanning electron microscopy
SSE: Secondary somatic embryo
MC: Moisture content
